# Mast cell exosomes promote lung adenocarcinoma cell proliferation – role of KIT-stem cell factor signaling

**DOI:** 10.1186/s12964-014-0064-8

**Published:** 2014-10-14

**Authors:** Hui Xiao, Cecilia Lässer, Ganesh Vilas Shelke, Juan Wang, Madeleine Rådinger, Taral Rameshchand Lunavat, Carina Malmhäll, Li Hui Lin, Jia Li, Li Li, Jan Lötvall

**Affiliations:** Department of Laboratory Medicine, Shanghai First People’s Hospital, Shanghai Jiao Tong University, Shanghai, China; Krefting Research Centre, Department of Internal Medicine, Sahlgrenska Academy, University of Gothenburg, Gothenburg, Sweden

**Keywords:** Exosomes, Extracellular vesicles, KIT, Lung cancer, Mast cell, Transfer

## Abstract

**Background:**

Human cells release nano-sized vesicles called exosomes, containing mRNA, miRNA and specific proteins. Exosomes from one cell can be taken up by another cell, which is a recently discovered cell-to-cell communication mechanism. Also, exosomes can be taken up by different types of cancer cells, but the potential functional effects of mast cell exosomes on tumor cells remain unknown.

**Methods and results:**

Exosomes were isolated from the human mast cell line, HMC-1, and uptake of PKH67-labelled exosomes by the lung epithelial cell line, A549, was examined using flow cytometry and fluorescence microscopy. The RNA cargo of the exosomes was analyzed with a Bioanalyzer and absence or presence of the *c-KIT* mRNA was determined by RT-PCR. The cell proliferation was determined in a BrdU incorporation assay, and proteins in the KIT-SCF signaling pathway were detected by Western blot. Our result demonstrates that exosomes from mast cells can be taken up by lung cancer cells. Furthermore, HMC-1 exosomes contain and transfer KIT protein, but not the *c-KIT* mRNA to A549 cells and subsequently activate KIT-SCF signal transduction, which increase cyclin D1 expression and accelerate the proliferation in the human lung adenocarcinoma cells.

**Conclusions:**

Our results indicate that exosomes can transfer KIT as a protein to tumor cells, which can affect recipient cell signaling events through receptor-ligand interactions.

## Introduction

Exosomes are nano-sized membrane vesicles (30–100 nm) constitutively released by various cells, such as B lymphocytes [1], dendritic cells [2,3], mast cells [4], natural killer (NK) cells [5], intestinal epithelial cells [6], tumor cells [7,8], and neuronal cells [9]. These extracellular vesicles have a lipid bilayer structure, and carry multiple proteins and RNA molecules [10]. Exosomes are known to shuttle their cargo, including proteins and lipids between cells [11], and in addition, we showed in 2007, that exosomes also shuttle functional RNA from one cell to another [12]. Importantly, the transfer of these molecules can change a recipient cell phenotype in many ways, and the exosome-mediated signal from the original cell may be different under different circumstances [13]. Exosomes are present in all human body fluids so far investigated, including saliva [14], blood plasma [15], breast milk [16], cerebrospinal fluid [17] and urine [18], and are abundant in the tumor microenvironment [19,20], indicating their importance for tumor biology.

The human *c-KIT* oncogene codes for the protein mast/stem cell growth factor receptor Kit (KIT), a member of the tyrosine kinase family of growth receptors [21]. KIT is expressed on a variety of hematopoietic cells, such as mast cells and bone marrow progenitor cells. Stem cell factor (SCF) dependent activation of KIT is critical to maintain homeostasis and function of mast cells [22]. In clinical lung cancer research, it has been shown that non-small cell lung cancer more rapidly leads to death if the tumor is KIT positive [23]. For example, if tumors are positive for KIT at the time of surgery, the disease is associated with short term survival, compared to those that are KIT negative [24]. In addition, co-expression of KIT and other tumor-promoting molecules such as EGFR tend to increase mortality further [25]. During some circumstances it is less clear how tumor cells become KIT positive, but one possibility is that non-tumor cells in the tumor microenvironment could shuttle such molecules between cells [26].

Tumors also harbor many other cells beside tumor cells, including inflammatory cells such as dendritic cells and mast cells [27,28], as well as fibroblasts and endothelium [29,30]. Furthermore, co-cultures of mast cells and non-small cell lung cancer leads to increased proliferation of the cancer cells both *in vitro* and *in vivo* [31]. In this study we therefore hypothesized that KIT could possibly be transferred to tumor cells via exosomes from one or several of the surrounding cells. To test this, we used a mast cell line (HMC-1) constitutively expressing the active form of the KIT receptor, and a non-small cell cancer lung epithelial tumor cell line (A549), to determine whether KIT can be transferred from mast cells to the epithelial cancer cell via exosomes, and whether those exosomes can influence the function of the recipient cell.

## Materials and methods

### Cell cultures

The lung adenocarcinoma cell line, A549, was obtained from the ATCC and the human mast cell line, HMC-1 (Dr Joseph Butterfield, Mayo Clinic, Rochester, MN, USA) was a kind gift from professor Gunnar Nilsson at the Karolinska Institute, Stockholm, Sweden. Control exosomes were derived either from the mouse embryonic fibroblast cell line, NIH 3T3 (Cell lines service, Eppelheim, Germany), or the human embryonic kidney 293 cell line, HEK 293 (from ATCC and a kind gift from Jonas Nilsson at the Sahlgrenska University Hospital, Gothenburg, Sweden). HMC-1 cells were maintained in Iscove’s modified Dulbecco’s medium (IMDM; HyClone Laboratories, Inc., Logan, UT, USA) supplemented with 10% exosome-depleted fetal bovine serum (FBS), 100 units/ml penicillin, 100 μg/ml streptomycin, 2 mM L-glutamine and 1.2 mM alpha-thioglycerol (all reagents were from Sigma-Aldrich, St Louis, MO, USA). NIH 3T3 cells were maintained in Dulbecco's modified Eagle medium (DMEM; HyClone Laboratories) and HEK 293 cells were maintained in Eagle's Minimum Essential Medium (EMEM, HyClone Laboratories), both medium were supplemented with 10% exosome-depleted FBS, 100 units/ml penicillin, 100 μg/ml streptomycin, 2 mM L-glutamine and 110 μg/ml sodium pyruvate (Sigma-Aldrich). The exosome-depleted FBS for the HMC-1, HEK 293 and NIH 3T3 cell cultures, was obtained by ultracentrifugtion at 120,000 × g for 18 hours using a Ti45 rotor (optima L-90 k Ultracentrifuge, Beckman Coulter, Brea, CA, USA). A549 cells were routinely maintained in DMEM/F-12 K medium (HyClone Laboratories, Inc.) supplemented with 10% FBS, 100 units/ml penicillin and 100 μg/ml streptomycin. All cells were cultured at 37°C in a humidified atmosphere of 5% CO_2_.

### Isolation of exosomes

Exosomes were isolated from the supernatant of HMC-1, HEK 293 and NIH 3T3 cells by differential centrifugation and a filtration step as described by Lässer *et al.* [32]. In brief, cell supernatant were harvested, centrifuged at 300 × g for 10 minutes to eliminate cells and at 16,500 × g for 20 minutes, followed by filtration through 0.2 μm filter (Sarstedt, Numbrecht, Germany) to eliminate cellular debris and larger vesicles. Exosomes were pelleted by ultracentrifugation at 120,000 × g for 70 minutes. Exosomes were measured for their protein content using the BCA protein assay kit (Thermo Scientific Pierce, Rockford, IL, USA).

### Electron microscopy

Isolated exosomes from HMC-1 cells were resuspended in PBS, and loaded onto UV-light pre-treated formwar/carbon-coated nickel grids (Ted Pella Inc., Redding, CA, USA). The exosomes were pre-fixed in 2% paraformaldehyde, before immuno-staining with anti-human CD63 antibody (BD Biosciences, San José, CA, USA). The exosomes were then immuno-stained with a 10 nm gold-labelled secondary antibody (Sigma-Aldrich) prior to being post- fixated in 2.5% glutaraldehyde and contrasted in 2% uranyl acetate. Preparations were examined in a LEO 912AB Omega electron microscope (Carl Zeiss Jena GmbH, Eching, Germany).

### Uptake of HMC-1 exosomes by A549 cells

To monitor uptake kinetics, exosomes derived from HMC-1, as well as the control cells HEK 293 and NIH 3T3 cells, were labelled with the green fluorescent dye PKH67 (Sigma-Aldrich) according to the adjusted protocol outlined in Lässer *et al.* [14]. Briefly, 20 μg of the PKH67-stained exosomes were washed five times using 300 kDa Vivaspin filters (Sartorius AG, Göttingen, Germany) to remove excess dye and then added to 2 × 10^5^ A549 cells in culture. Cells were harvested at different time points (1, 2, 4, 8, 12, 24 and 48 hours), washed three times and analyzed by flow cytometry using a BD FACSAria (BD Biosciences). Additionally, cells were visualized at 8 or 12 hours with a fluorescence microscopy (Zeiss Axioplan 2 microscope, Carl Zeiss) or at 4 hours with confocal laser scanning microscope (LSM 700, Carl Zeiss). As control for non-specific labelling of the cells, PBS was PKH67 stained, washed and added to the cells in a parallel experiment. For analysis with flow cytometry, the A549 cells were washed twice with PBS, treated with 0.25% trypsin-EDTA solution (Sigma-Aldrich) to detach the cells and washed twice with 1% FBS in PBS before acquired in a BD FACSAria flow cytometry running BD FACS Diva version 6.0 Software (BD Biosciences) and analyzed with the FlowJo Software (Tree Star Inc., Ashland, OR, USA). For microscopy, the cells were washed twice with PBS, fixed with 4% formaldehyde solution for 15 minutes and washed again twice with PBS. For the fluorescence microscopy the cells were mounted with Vectashield (Vector Laboratories Inc., Burlingame, CA, USA) supplemented with 3% 7-Aminoactinomycin (7-ADD; BD Biosciences) to label cell nuclei and for confocal microscopy cells were mounted with ProLong® Gold Antifade Mountant containing 4',6-diamidino-2-phenylindole (DAPI; Life Technology).

### Transfer of KIT protein and c-KIT mRNA to A549 cells

To determine if the KIT protein could be transferred via exosomes, HMC-1 exosomes (80 μg) were added to A549 cells (4 × 10^5^) and the cells were incubated for 24 hours. The A549 cells were harvested, washed and the total proteins were isolated using RIPA buffer (Cell Signaling Technology, Danvers, Massachusetts, USA). As a control, cells were treated with only media. The presence of KIT was determined using Western blot.

To determine if the *c-KIT* mRNA could be transferred via exosomes, HMC-1 exosomes (80 μg) were added to A549 cells (4 × 10^5^). The cells were harvested at different time points (30 minutes, 2, 4, 24 and 48 hours), washed and isolation of total RNA was performed. Presence of *c-KIT* mRNA was determined using reverse transcription polymerase reaction (RT-PCR).

### RT-PCR

Total RNA was isolated using miRCURY™ RNA Isolation Kit (Exiqon¸Vedbaek, Denmark) according to the manufacturer’s protocol and as previously described [33]. Detection and quantity of RNA was determined using a Bioanalyzer and RNA 6000 Nano chips according to the manufacturer’s protocol (Agilent Technologies, Santa Clara, CA, USA).

For examination of the *c-KIT* gene expression, 500 ng of total RNA was converted into cDNA using RT^2^ first Strand Kit (Qiagen, Hilden, Germany) according to the manufacturer’s recommendations. For PCR amplification 1 μl of diluted cDNA (<500 ng) was used as a template to make 50 μl reactions containing the reagents from HotStarTaq Master Mix (Qiagen). KiCqStart™ primer pairs (Sigma-Aldrich) used were as follows: GAPDH (forward) 5'-CTTTTGCGTCGCCAG-3', (reverse) 5'-TTGATGGCAACAATATCCAC-3'; *c-KIT* (forward) 5'-ACAAAACCAGAAATCCTGAC-3', (reverse) 5'-CAGTTCCTGGACAAAAATACC-3'. The length of the expected amplicon for *c-KIT* and *GAPDH* were 109 and 139 base pair, respectively. PCR amplification involved 30 cycles at 94°C for 30 seconds, 55°C for 45 seconds, and 72°C for 30 seconds. PCR products were resolved on a 1.5% agarose gel (by loading 5 μl of the PCR product) and detected utilizing Gel Star® Nucleic Acid Gel Stain (Lonza, Rockland, ME, USA) and a VersaDoc 4000 MP (Bio-Rad Laboratories, Hercules, CA, USA).

### Reverse migration assay

To evaluate the functional effect on lung epithelial cells (A549) induced by HMC-1 derived exosomes, reverse migration assay was performed using a 46 well Boyden chamber (Neuroprobe Inc.). Briefly, A549 cells (32,500 cells/well) were added in the lower chamber and allowed to adhere onto the gelatin (0.1%) coated polycarbonate membrane by inverting the Boyden chamber upside down (Neuroprobe Inc.). After 3 hours of incubation the assembly was placed in correct orientation and exposed to various HMC-1 exosomes dosage on the upper side of the membrane for 12 hours. Migrating cells towards the upper side of membrane were fixed in ethanol, and stained with Giemsa (Histolab, Gothenburg, Sweden) and images were acquired using a light microscope (Zeiss Axioplan, Germany). Adhered cells, present on lower side of membrane (non-migrated side), were wiped out carefully before imaging.

### Detection of cell proliferation

Bromodeoxyuridine (BrdU) is incorporated into the newly synthesized DNA strands of actively proliferating cells. We therefore determined cell proliferation using a BrdU labeling ELISA kit (Calbiochem, San Diego, CA, USA) according to the manufacturer’s protocol. Shortly A549 cells were seeded at the density of 1 × 10^5^ in a 96-well plate and incubated overnight to allow cells to attach to the plate. Cells received BrdU (20 μl) and 20 μg of either the HMC-1 exosomes or the control exosomes (isolated from NIH 3T3 cells) at the same time. After 4 hours of incubation the absorbance was obtained at dual wavelength (450 nm and 595 nm) with a spectrophotometer (Spectra Max; Molecular Devices, Sunnyvale, CA, USA). The results were normalized as percent of control.

### Western blot analysis

The cellular and exosomal proteins were separated by SDS-PAGE, transferred to nitrocellulose membrane (Bio-Rad Laboratories), blocked (5% BSA-TBST; bovine serum albumin in TBS tween 20) for 2 hours. The membrane were then incubated with the following primary rabbit anti-human antibodies: anti-CD81 (Santa Cruz Biotechnology, Dallas, TX, USA), anti-calnexin (Santa Cruz Biotechnology), anti-KIT (Abcam, Cambridge, UK), anti-SCF (Santa Cruz Biotechnology), anti-PI3K and anti-p-PI3K P85(T458)/P55(T199), anti-AKT and anti-p-AKT(T308), anti-GSK3β and anti-p-GSK3β(S9), and anti-cyclin D1 and anti-p-cyclin D1(T286) and primary mouse anti-human antibody; TSG101 (Abcam) diluted in 5% BSA -TBST at 4°C overnight. All antibodies were purchased from Cell Signaling Technology Inc., (Danvers, MA, USA) unless stated otherwise. The membrane was washed 3 × 5 minutes before incubation with the secondary antibody for 2 hours. The secondary antibodies used were goat F(ab)2 anti-rabbit IgG or donkey anti-mouse IgG (HRP conjugated, Harlan Sera-Lab, Loughborough, UK) diluted in 1% BSA-TBST. The membrane was washed 3 × 5 minutes, before being analyzed with the Amersham™ECL Plus™ Western Blotting Detection System (GE Healthcare, Buckinghamshire, UK) and a VersaDoc 4000 MP (Bio-Rad Laboratories). To normalize protein loading, identical loaded samples were probed for GAPDH. Relative intensity was calculated as follows for p-PI3K, p-AKT and p-GSK3β; (phosphorylated protein/GAPDH)/(total protein/GAPDH), and for cyclin D1; cyclin D1/GAPDH.

### Statistical methods

The statistical analyses were performed using SPSS 16.0 statistical software (SPSS, Chicago, IL, USA). Student’s *t*-test was used to analyze the difference between two groups, and one-way ANOVA followed by Dunnett’s test or Kruskal-Wallis test followed by Dunn´s multiple comparisons test was employed for the comparisons among three or more groups. To estimate correlation, a Spearman ranked correlation test was performed. Data are presented as the mean ± SEM for all statistical tests. Results were considered to be statistically significant when p ≤0.05.

## Results

### Identification of mast cell exosomes and their effect on lung adenocarcinoma cells

**Figure 1:**
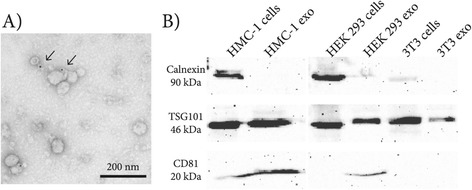
**Identification and characterization of HMC-1 exosomes.** Exosomes were isolated using differential centrifugation. **A)** The electron micrographs of the exosomes revealed rounded structures with a size of approximately 30–100 nm. Arrows indicate 10-nm gold-labeled anti-CD63. The scale bar indicates 200 nm. **B)** Western blot analysis of exosomes derived from HMC-1 cells supernatant shows presence of the common exosome proteins, CD81 and TSG101, but absence of the endoplasmic reticulum protein, calnexin. Also control exosomes isolated from NIH 3T3 and HEK 293 cells were positive for TSG101 and CD81, but negative for calnexin. Cells were used as a control.

After isolation of exosomes from the supernatant of cultured HMC-1 cells, using ultracentrifugation, CD63 positive vesicular round structures could be visualized by electron microscopy (Figure 1A). The protein fraction isolated from the pellet after ultracentrifugation was positive for the traditional exosome markers CD81 and TSG101, while negative for a endoplasmic reticulum protein, calnexin (Figure 1B), confirming our previous data showing that HMC-1 cells can release exosomes [12,34,35]. Furthermore, exosomes isolated from HEK 293 and NIH 3T3 cells were also positive for the exosomal markers and negative for the endoplasmic reticulum protein calnexin (Figure 1B). These exosomes were therefore considered to be appropriate to be used as control exosomes in this study.

**Figure 2:**
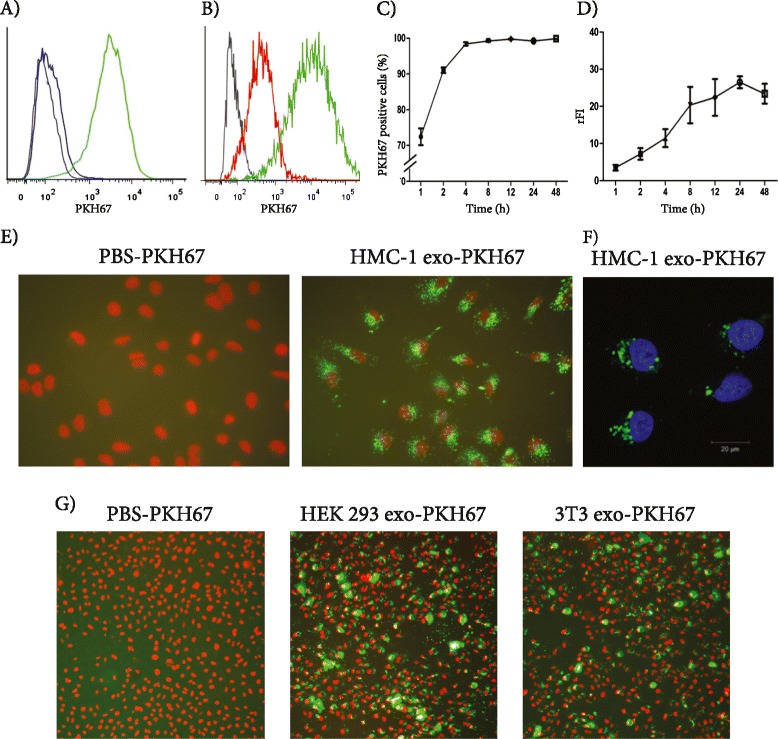
**Uptake of human mast cell exosomes by human lung adenocarcinoma cells.** Twenty microgram of the PKH67-labelled HMC-1, HEK 293 or NIH 3T3 exosomes, or a PKH67-PBS control were added per 2 ×10^5^ A549 cells and incubated at 37°C or 4°C for 1–48 hours. The uptake of the fluorescently labelled exosomes by A549 was detected with flow cytometry (at all the time points), fluorescence microscopy (at the 12 hour time point) and confocal microscopy (at the 4 hour time point). **A)** Representative graph of uptake at 4 hours at 37° detected with flow cytometry. Black curve, control cells; blue curve, PKH67-PBS control; green curve, PKH67-labelled HMC-1 exosomes. **B)** Representative graph of uptake at 8 hours detected with flow cytometry. Black curve, control cells; red curve, PKH67-labelled HMC-1 exosomes at 4°C; green curve, PKH67-labelled HMC-1 exosomes at 37°C. **C** **and** **D)** Percent positive cells and relative fluorescence intensity (rFI) data for all time points determined with flow cytometry are shown as mean ± SEM (n =3). **E)** Uptake of PKH67-PBS control and PKH67-labelled exosomes at 37°C imaging with fluorescence microscopy. Nuclei were stained with 7-AAD (red). **F)** Uptake of PKH67-labelled exosomes at 37°C imaging with confocal microscopy (bar is indicating 20 μm). Nuclei were stained with DAPI (blue). **G)** Uptake of PKH67-labelled NIH 3T3- and HEK 293-derived exosomes at 37°C imaging with fluorescence microscopy. Nuclei were stained with 7-AAD (red).

To examine whether exosomes from human mast cells can be taken up by lung cancer cells, the HMC-1 exosomes were labeled with PKH67 dye and added to cultures of A549 cells. Flow cytometry analysis (Figure 2A) showed an increased fluorescence intensity of the A549 cells after the addition of mast cell-derived exosomes, indicating cellular uptake. This uptake was reduced at 4°C, arguing that the uptake was an active process (Figure 2B). The fluorescence of A549 cells was obvious already two hours after addition of the stained exosomes, and increased over time (Figure 2C and D), indicating initiation of uptake of the exosomes by A549 cells. The uptake of the fluorescent exosomes by the A549 cells was also visualized using fluorescence microscopy (Figure 2E) and confocal microscopy (Figure 2F). Additionally, fluorescence microscopy demonstrated that control exosomes isolated from HEK 293 and NIH 3T3 cells were taken up by A549 cells in a similar manner as HMC-1 exosomes (Figure 2G). This verifies their validity to be used as control exosomes in the later functional experiments.

**Figure 3:**
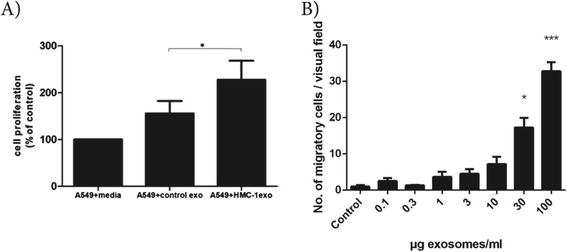
**Mast cell-derived exosomes induce proliferation and migration in A549 cells. (A)** BrdU cell proliferation assays were used to detect proliferating A549 cells after co-culturing for 4 hours with HMC-1 exosomes or control exosomes (derived from NIH 3T3 cells). *P <0.05. **(B)** A549 cells were added to the lower chamber of a Boyden chamber (32 500 cells/well). To the upper chamber 30 μl of the different doses of HMC-1 exosomes were added. Media was used as a control. After 12 hours the number of cells migrated to the lower chamber of the 8 μm pore-sized membrane were analyzed by taking photos and counting the number of cells per visual field. Kruskal-Wallis test followed by Dunn’s multiple comparisons test were used to determine significant differences where all concentrations were only compared to the control. Spearman’s rank correlation coefficient was 0.91 (p <0.0001). p-values; * <0.05, *** <0.001.

To determine whether mast cell exosomes can influence lung adenocarcinoma, both proliferation and migration was assessed in the A549 cells in the presence of HMC-1 exosomes. Firstly, HMC-1 exosomes were added to A549 cells in culture for 4 hours in the presence of BrdU to evaluate their capacity to influence proliferation. The incorporation of BrdU was significantly increased by 142% in the presence of HMC-1 exosomes compared to the medium control and by 38% compared to the control exosomes, as quantified by an ELISA kit (Figure 3A). Secondly, the A549 cells were seeded on the membrane of the lower chamber with different doses of HMC-1 exosomes present in the upper chamber to evaluate the exosomal capacity to induce migration. Significantly more cells migrated into the upper chamber in a dose response dependent manner in the presence of HMC-1 exosomes, with the highest doses being statistically significant compere to the control and with a correlation coefficient of 0.91 (p <0.0001) (Figure 3B).

### The presence of KIT protein in mast cell exosomes and their shuttling to lung adenocarcinoma cells

**Figure 4:**
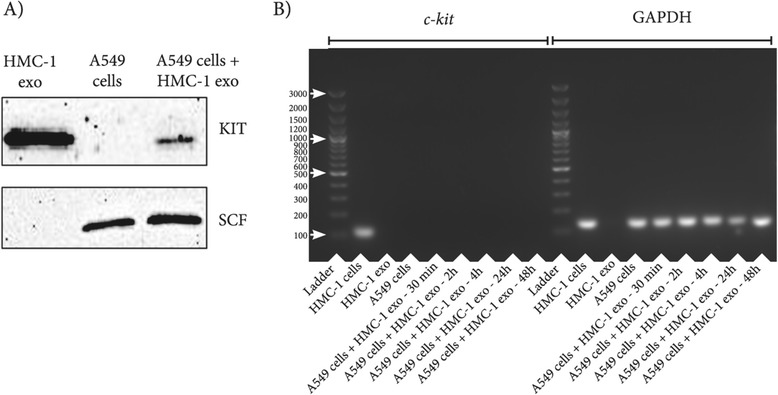
**HMC-1 exosomes transfer KIT protein to A549 cells.** Eighty microgram of HMC-1 exosomes were added per 4 ×10^5^ A549 cells and incubated at 37°C for 24 hours for the Western blot experiments **(A)** and for 30 minutes - 48 hours for the RT-PCR experiments **(B)**. **A)** Western blot confirmed the presence of the KIT protein in HMC-1 exosomes, but not in the A549 cells. SCF expression was detected in A549 cell but not in the HMC-1 exosomes. SCF and KIT were both detected in A549 cells 24 hours after the addition of HMC-1 exosomes. **B)** RT-PCR showed that *c-KIT* mRNA was present in HMC-1 cells, whereas it was absent in both HMC-1 exosomes and A549 cells. GAPDH mRNAs were detected as positive control and were detected in all samples except in HMC-1 exosomes.

The presence of the KIT protein in HMC-1 exosomes is illustrated in Figure 4A. By contrast, the A549 cells had no detectable KIT, as determined by Western blot. However, when HMC-1 exosomes were incubated with the A549 cells for 24 hours, KIT could be detected in the A549 cells by Western blot (Figure 4A). Stem cell factor (SCF), the ligand for KIT, was however present in the A549 cells, but absent in HMC-1 exosomes. By contrast, we were unable to detect any *c-KIT* mRNA in the exosomes, but only in the HMC-1 cells (Figure 3B). Furthermore, the *c-KIT* mRNA was not detected in the A549 cells at any of the time points analyzed. GAPDH were used as positive controls, and GAPDH mRNA was present in all samples, except in the HMC-1 exosomes (Figure 3B). Our identification of KIT protein, but not *c-KIT* mRNA, in the HMC-1 exosomes, and the transfer of the protein to lung adenocarcinoma cells, suggests that the presence of KIT in the A549 cells after exosomes depend on transfer of KIT protein, rather than transfer of its mRNA (Figure 4A and B).

### Mast cell exosomes activate the KIT-SCF signaling pathway in recipient lung adenocarcinoma cells

**Figure 5:**
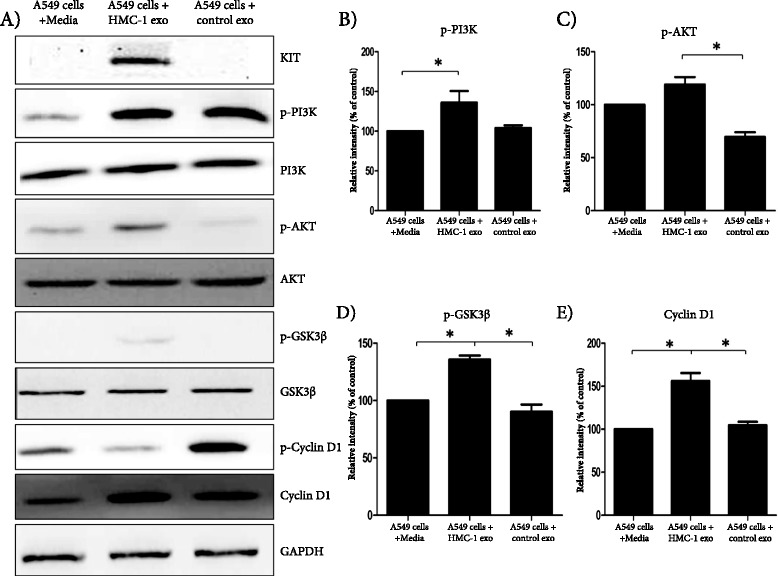
**Mast cell-derived exosomes can activate the KIT-SCF signaling pathway in A549 cells.** A549 cells treated with exosomes from control cells (HEK 293) or HMC-1 cells were analyzed using Western blot. Phosphorylated and total PI3K, AKT, GSK3β and cyclinD1 were measured by Western blot and to normalize protein loading, samples were also probed for GAPDH. **(A)**. Relative intensity was calculated as follows for p-PI3K **(B)**, p-AKT **(C)** and p-GSK3β **(D)**; (phosphorylated protein/GAPDH)/(total protein/GAPDH), and for cyclin D1 **(E)**; cyclin D1/GAPDH. All the above data are representative of three independent experiments (n =3). *P <0.05 and **P <0.01.

Next, we sought to determine whether transfer of KIT by HMC-1 exosomes could influence A549 intracellular KIT-associated pathways, proposing a molecular explanation for the observed enhanced proliferation seen in Figure 3A. As shown in Figure 5A-E, we indeed found that PI3K and its downstream targets, AKT, and GSK3β showed increased phosphorylation in A549 cells when HMC-1 exosomes were added, compared to media alone or control exosomes. However, the total amounts of these three proteins were unchanged according to the Western blot analysis. When PI3K is phosphorylated and thus activated it phosphorylates AKT, which in turn inactivates GSK3β by phosphorylating the S^9^ residue on GSK3β. Cyclin D1 is a cell-cycle regulator downstream of GSK3β and is inhibited by GSK3β. Therefore an increase in deactivation of GSK3β by phosphorylation will lead to an increase in cyclin D1, which will result in cells moving from the G1 phase into the S phase of the cell cycle. Importantly, cyclin D1, was indeed increased in A549 cells after the addition of HMC-1 exosomes, which then can be associated to increased proliferation of the lung adenocarcinoma cells. When cyclin D1 is phosphorylated it is transported out of the nucleus and degraded, and as shown in Figure 5A, this inactivated stage is attenuated by HMC-1 exosomes, which thus further can enhance the activation of cyclin D1.

These experiments together indicate that KIT positive exosomes from HMC-1 cells have the capability to induce PI3K/AKT signaling and influence cell proliferation in A549 cells.

## Discussion

Tumor tissues include many different cells, and little is known how they interact in a clinical situation to influence cancer development. It has been suggested that exosomes and other extracellular vesicles can shuttle many molecules between cells, which is a process likely to be very active in cell-to-cell signaling in tumors. Tumor tissue also contains many inflammatory cells, and it is known that mast cells can be part of the tumor microenvironment in lung tumors such as non-small cell lung cancer. However, little is known how mast cells might act to specifically promote cancer proliferation. Here, we found that exosomes derived from the human mast cell line, HMC-1, contain the receptor KIT, which can be transferred to lung adenocarcinoma cells by exosomes. In particular, we show that mast cells release exosomes with typical exosome markers such as TSG101 and CD81. The mast cell exosomes are rapidly taken up by lung adenocarcinoma cells (A549 cells), a process that peaks after approximately 12 hours. Importantly, the exosomes released by human mast cells contain the natural growth factor receptor KIT, but not *c-KIT* mRNA. Addition of mast cell exosomes containing KIT enhance proliferation and migration of the lung adenocarcinoma cells, and enhance the KIT-SCF signaling pathway activity. Overall, these data suggest that exosomes from mast cell can enhance proliferation of lung adenocarcinoma cells, putatively by enhancing KIT-SCF signaling in tumor cells.

In this study, we confirm the ability of the mast cell line HMC-1 to release exosomes into their microenvironment [34], and extend these findings by demonstrating their ability to be taken up by human lung adenocarcinoma cells (A549 cells). The observed uptake is rapid, as more than 70% of cells have taken up some exosomes already after one hour, and after four hours almost all cells are positive for exosomes. However, the uptake continues over time, measured as relative fluorescent intensity, and peaks at 12–24 hours after addition of exosomes. This time course of uptake is slightly faster then what we have previously reported [34].

Less is known about the functionality of mast cell exosomes. In the current experiments, we have described the presence of the KIT tyrosine kinase receptor in mast cell exosomes, a molecule that sometimes, but not always, is expressed in lung tumor tissues [36]. Importantly, we were unable to detect the *c-KIT* mRNA in the exosomes, suggesting that any functional shuttling of this molecule between cells is at the protein level. KIT was present in the lung adenocarcinoma cell line after addition of mast cell exosomes, documented using Western blot analysis. KIT has been considered to be a marker of progenitor cells [37] and can be more expressed in preneoplastic tissue and tumors, such as gastrointestinal stromal tumor (GIST) [38], small cell lung cancer (SCLC) [39], colorectal cancer [40,41] and pancreatic neoplasms [42]. KIT has also been implied in non-small cell lung cancer mortality, indicating that it may influence tumor growth and metastasis [23].

In the present study we confirm that addition of KIT-containing mast cell exosomes can enhance the proliferation of lung adenocarcinoma cells, shown as uptake of BrdU. A similar finding was recently published in which patient tumor exosomes containing KIT protein could transfer that protein to gastrointestinal stromal tumor cells and smooth muscle cells *in vitro*, which enhanced signs of invasiveness [26]. In our study, we have also included an appropriate exosome control in all experiments, and provided evidence that the KIT containing mast cell exosomes can enhance the KIT-SCF signaling pathways intracellularly.

We could here detect enhanced KIT-SCF signaling pathways, by up-regulation of phosphorylated PI3K as well as AKT and GSK3β. These data suggest, collectively, that the KIT containing mast cell exosomes indeed can activate cascades downstream from the tyrosine kinase receptor KIT. We therefore propose that the tumor cell-produced SCF can help tumor growth via a PI3K dependent pathway, if mast cell KIT-containing exosomes are delivered to the cells. Mast cell exosomes carrying KIT may bind SCF molecules produced by the tumor cells, and activate the cell in an autocrine fashion via PI3K and AKT. It is known that AKT can enhance tumor growth, by phosphorylating and inactivating GSK3β. This can in turn lead to a decrease in the nuclear exclusion of cyclin D1, which thereby allow the molecule to functioning as a cell cycling promoter by driving the cells from G1 phase to S phase [43,44]. Overall, our data imply that mast cell KIT-containing exosomes can influence the PI3K signaling pathway in recipient tumor cells.

There are of course shortcomings with *in vitro* studies such as our, as results may or may not be applicable for clinical disease. However, in a previous clinical study of non-small cell lung tumors, we found that KIT positivity was associated with higher mortality [23].

Our current series of experiments have demonstrated the release of KIT-containing exosomes from a human mast cell line, and shows that these exosomes can be taken up by lung adenocarcinoma cells. This leads to enhanced proliferation in recipient tumor cells, by the activation of the PI3K signaling pathway. Future work can highlight the molecular mechanisms leading to the release of exosomes from mast cells, and their uptake into tumor cells. Further, it is essential to understand the diverse possibilities of exosome-based intercellular communication among many different types of cells in the tumor microenvironment.

## Abbreviations

SCF: Stem cell factor
FBS: Fetal bovine serum
PI3K: Phosphatidylinositol-3-kinase
GSK3β: Glycogen synthase kinase-3 β
GAPDH: Glyceraldehyde 3-phosphate dehydrogenase
